# Clinical and Genetic Analysis of Dehydrated Hereditary Stomatocytosis: A Case Report

**DOI:** 10.1002/ccr3.71005

**Published:** 2025-10-06

**Authors:** Chen Ming, Shiyuan Wu, Rui Pan

**Affiliations:** ^1^ Department of Pediatrics Xiangyang Central Hospital, Affiliated Hospital of Hubei University of Arts and Science Xiangyang China; ^2^ Clinical Research Center for Pediatric Diseases and Rare Diseases Xiangyang China

**Keywords:** case report, dehydrated hereditary stomatocytosis, genetic testing, hemolytic anemia, PIEZO1 mutation

## Abstract

Dehydrated hereditary stomatocytosis (DHS) is a rare autosomal dominant hemolytic anemia caused by abnormal erythrocyte ion permeability, most often due to *PIEZO1* mutations. We report the case of a 15‐year‐old male with elevated indirect bilirubin and mild anemia. Peripheral smear showed target cells, and whole‐exome sequencing identified a heterozygous *PIEZO1* mutation (c.7367G>A, p.R2488Q), confirming DHS. Family testing revealed paternal inheritance. The patient remains asymptomatic and is managed conservatively with regular follow‐up. This case highlights the importance of genetic testing for early diagnosis and counseling in rare hemolytic disorders.

## Graphical abstract

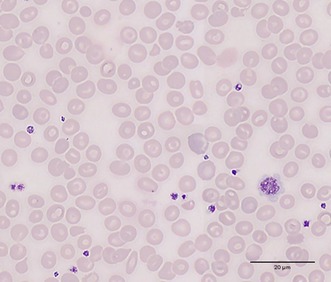

**Summary**

- Dehydrated hereditary stomatocytosis is a rare inherited hemolytic anemia.
- Genetic testing, particularly identification of PIEZO1 mutations, is essential for accurate diagnosis, guiding management, and avoiding unnecessary splenectomy.

## 1 Introduction

Dehydrated hereditary stomatocytosis (DHS) is a rare autosomal dominant hemolytic anemia characterized by red blood cell (RBC) dehydration due to altered membrane cation permeability. This leads to cell dehydration, decreased deformability, and chronic hemolysis. DHS is primarily associated with mutations in genes encoding ion channels, such as PIEZO1 and KCNN4 [1, 2], which play crucial roles in maintaining RBC volume and electrolyte balance. The clinical presentation of DHS is highly variable, ranging from asymptomatic individuals to those with significant anemia, jaundice, and splenomegaly, making diagnosis challenging.

The purpose of this case report is to highlight the importance of genetic testing in diagnosing DHS, particularly in patients presenting with unexplained hemolysis and hyperbilirubinemia. By sharing our findings, we aim to contribute to the limited literature on DHS and emphasize the need for increased awareness among clinicians to consider this rare condition in differential diagnoses.

We present the case of a 15‐year‐old male who exhibited elevated indirect bilirubin levels during a routine health check‐up. Subsequent investigations led to the identification of a pathogenic PIEZO1 gene mutation, confirming the diagnosis of DHS. This case emphasizes the critical role of genetic analysis in the early and accurate diagnosis of rare hematological disorders. The studies were approved by the Ethics Committee of Xiangyang Central Hospital (protocol code 2024‐110).

## 2 Case Presentation

### 2.1 Chief Complaints

The patient, a 15‐year‐old male, presented to our hospital for further evaluation of elevated bilirubin levels, primarily indirect bilirubin, identified during a routine health check‐up. Previous diagnostic tests at another hospital had not clarified the cause, prompting referral to our institution for further evaluation.

### 2.2 History of Present Illness

In December 2023, the patient's routine health check revealed elevated bilirubin levels, predominantly indirect bilirubin. Although relevant diagnostic tests were conducted at an external hospital, the cause was not identified, prompting the patient's transfer to our hospital for further assessment. Laboratory results at admission revealed:

- Complete blood count: Hemoglobin (Hb) 130 g/L, red blood cell (RBC) count 6 × 10^12^/L, mean corpuscular volume (MCV) 65.4 fL, mean corpuscular hemoglobin (MCH) 21.7 pg, mean corpuscular hemoglobin concentration (MCHC) 332 g/L, Reticulocyte count 3.7%.
- Liver function: Total bilirubin (TBil) 44.3 μmol/L, direct bilirubin (DBil) 9.6 μmol/L, indirect bilirubin (IBil) 34.7 μmol/L, alanine aminotransferase (ALT) 5 U/L, aspartate aminotransferase (AST) 11 U/L.
- Hepatitis B virus (HBV): Surface antigen, surface antibody, e‐antigen, e‐antibody, core antibody, and HBV DNA were all negative.
- Iron studies: Serum iron 44.5 μmol/L, total iron binding capacity (TIBC) 47.8 μmol/L, Ferritin 287.8 ng/mL.
- Coagulation function: Prothrombin time (PT) 14.8 s, fibrinogen (FIB) 1.51 g/L, activated partial thromboplastin time (aPTT) 43.7 s.
- Immunology: Antinuclear antibody (ANA) negative, anti‐smooth muscle antibody (SMA) negative, anti‐liver soluble antigen i antibody, anti‐soluble liver antigen/pancreas antibody, anti‐liver‐kidney microsomal antibody, anti‐mitochondrial antibody M2 subtype were all negative.
- Abdominal ultrasound: No space‐occupying lesions were observed in the liver, gallbladder, spleen, or pancreas.

### 2.3 Personal and Family History

The patient's father has a history of similar liver function abnormalities, while the mother's liver function is normal. There is no other notable family history of related diseases.

### 2.4 Physical Examination

The patient appeared generally well with no apparent abnormalities. There were no signs of jaundice, anemia, or hepatosplenomegaly. Other physical findings were normal.

### 2.5 Differential Diagnosis

The differential diagnosis included other forms of hereditary hemolytic anemia such as hereditary spherocytosis, as well as acquired hemolytic anemia due to autoimmune causes. The clinical presentation and lab findings were consistent with dehydrated hereditary stomatocytosis (DHS).

## 3 Methods

After considering the differential diagnoses, further diagnostic investigations were undertaken, including genetic testing and whole‐exome sequencing to confirm the diagnosis of dehydrated hereditary stomatocytosis (DHS).

### 3.1 Laboratory Examinations

**FIGURE 1:**
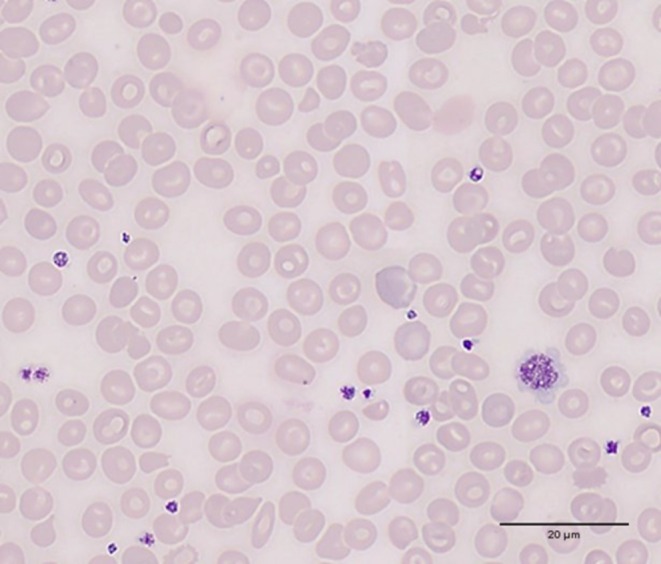
Morphology of erythrocytes in peripheral blood slices of patients with hereditary orocytosis due to mutations in the PIEZO1 gene (arrows showing typical stomatocytes).

Upon admission, further laboratory investigations revealed:

- Complete blood count: Hemoglobin (Hb) 119 g/L, mean corpuscular volume (MCV) 67.1 fL, reticulocyte count 4.73%.
- Liver function: Total bilirubin (TBil) 32.8 μmol/L, direct bilirubin (DBil) 16.8 μmol/L, indirect bilirubin (IBil) 16 μmol/L, alkaline phosphatase (ALP) 147 U/L, albumin (ALB) 44.9 g/L.
- Hemolysis panel: Coombs test negative; glucose‐6‐phosphate dehydrogenase (G6PD), red blood cell osmotic fragility, and hemoglobin electrophoresis were all normal; peripheral blood smear revealed 18.7% target cells (Figure 1).

### 3.2 Whole‐Exome Sequencing

Whole‐exome sequencing revealed a heterozygous mutation in the PIEZO1 gene at exon 16, position chr16:7367135G>A, NM_001142864.4.7367G>A, p.(R2488Q). This mutation has been previously reported in the literature and is associated with dehydrated hereditary stomatocytosis (DHS), a disorder that affects red blood cell volume regulation, typically presenting with mild anemia, jaundice, and splenomegaly. The p.(R2488Q) mutation is listed in ClinVar as a pathogenic variant (VCV000000580932.2). The allele frequency of this mutation in the gnomAD database v3.1.1 is low, indicating its rarity in the general population. Based on supporting evidence, this mutation has been classified as pathogenic, with functional impacts on red blood cell volume regulation and a consistent association with DHS clinical features. Family analysis showed that the patient's father also carries the same heterozygous PIEZO1 mutation, while the mother does not carry the mutation. This autosomal dominant inheritance pattern further confirms the pathogenicity of the PIEZO1 mutation and aligns with the genetic transmission pattern of DHS.

### 3.3 Final Diagnosis

The patient was ultimately diagnosed with Dehydrated Hereditary Stomatocytosis (DHS), caused by the PIEZO1 gene mutation (c.7367G>A: p.R2488Q), based on the clinical symptoms, laboratory findings, imaging studies, and genetic test results.

### 3.4 Follow‐Up

As the patient is currently asymptomatic and there is no evidence of severe hemolysis or acute symptoms, no pharmacological treatment was initiated. Hospitalization was not necessary. The patient was advised to undergo regular follow‐up visits to monitor hemoglobin levels, reticulocyte count, and liver function.

## 4 Discussion

Dehydrated hereditary stomatocytosis (DHS) is a rare form of hereditary hemolytic anemia characterized by red blood cell (RBC) dehydration due to altered ion transport across the erythrocyte membrane. The pathogenesis of DHS is complex and heterogeneous, involving mutations in various genes that affect ion channels and membrane transporters. Mutations in the PIEZO1 gene are among the most commonly identified causes, but mutations in other genes such as KCNN4 and ATP11C have also been implicated, indicating that DHS is a genetically diverse disorder [1, 3].

In this case, the patient presented with mild anemia and elevated indirect bilirubin levels, which are indicative of hemolysis. The peripheral blood smear showed the presence of target cells, a hallmark of RBC dehydration. The diagnosis of DHS was confirmed through whole‐exome sequencing, which identified a heterozygous mutation in the PIEZO1 gene (c.7367G>A, p.R2488Q). This mutation has been previously reported and is known to cause gain‐of‐function effects in the PIEZO1 channel, leading to increased cation permeability and subsequent RBC dehydration [4, 5].

The clinical presentation of DHS can be highly variable, ranging from asymptomatic individuals to those with severe hemolytic anemia and complications such as splenomegaly, cholelithiasis, and iron overload due to chronic hemolysis [6]. The variability in clinical manifestations suggests that other genetic, epigenetic, or environmental factors may modulate disease expression. For instance, co‐inheritance of other genetic variants or differences in the expression levels of interacting proteins could influence the severity of the disease [7].

The identification of a PIEZO1 mutation in this patient not only confirms the diagnosis but also has important clinical implications. PIEZO1 is a mechanosensitive ion channel that responds to mechanical stress by allowing the influx of cations, particularly calcium and sodium, into the cell [8]. Gain‐of‐function mutations enhance this activity, leading to an imbalance in ion homeostasis and cell dehydration. Understanding the specific genetic mutation allows for more precise counseling regarding disease prognosis and potential complications.

Differential diagnosis is critical in patients presenting with hemolytic anemia and abnormal RBC morphology. Conditions such as hereditary spherocytosis, hereditary elliptocytosis, and other forms of hereditary stomatocytosis must be considered [9]. Laboratory tests including osmotic fragility, ektacytometry, and genetic analyses are essential for accurate diagnosis. In this case, the negative Coombs test and normal G6PD activity helped exclude autoimmune hemolytic anemia and G6PD deficiency, respectively.

Management of DHS is primarily supportive, focusing on monitoring and treating complications. Regular follow‐up is necessary to assess hemoglobin levels, reticulocyte counts, bilirubin levels, and markers of iron status to detect iron overload early [10]. Iron chelation therapy may be required if ferritin levels indicate significant iron accumulation. Splenectomy is generally not recommended due to the risk of thrombosis, which may be heightened in DHS patients [11].

The patient's father was found to carry the same PIEZO1 mutation, consistent with an autosomal dominant inheritance pattern. This finding underscores the importance of family screening and genetic counseling. Relatives may be asymptomatic carriers or have mild symptoms that have gone unnoticed. Early identification allows for appropriate monitoring and management of potential complications.

Recent research has explored potential therapeutic targets for DHS. Modulators of the Gardos channel (encoded by KCNN4) and inhibitors of PIEZO1 are being investigated to restore ion homeostasis in affected RBCs [12, 13]. While these treatments are not yet clinically available, they represent promising avenues for future therapy. Gene therapy could also be a potential strategy, although challenges related to delivery and long‐term expression remain.

Moreover, the role of PIEZO1 extends beyond erythrocytes; it is expressed in various tissues and is involved in processes such as vascular development, blood pressure regulation, and lymphatic function [14]. Therefore, patients with PIEZO1 mutations may have subtle extramedullary manifestations that warrant further investigation. Long‐term studies are needed to fully understand the systemic implications of PIEZO1 dysfunction.

In conclusion, this case highlights the significance of considering DHS in patients with unexplained hemolysis and the critical role of genetic testing in establishing the diagnosis. A multidisciplinary approach involving hematologists, geneticists, and primary care providers is essential for optimal patient care. Continued research into the molecular mechanisms of DHS will hopefully lead to targeted therapies that can improve outcomes for patients with this rare condition.

## 5 Conclusion

Early genetic testing is crucial for diagnosing DHS, enabling appropriate management and informed family counseling.

## Author Contributions

**Chen Ming:** resources, software, supervision, validation, writing – original draft, writing – review and editing. **Shiyuan Wu:** formal analysis, funding acquisition, writing – original draft, writing – review and editing. **Rui Pan:** conceptualization, data curation, formal analysis, project administration.

## Ethics Statement

The studies were approved by the Ethics Committee of Xiangyang Central Hospital (protocol code 2024‐110).

## Consent

The patient's parents provided informed written consent for the publication of this case report.

## Conflicts of Interest

The authors declare no conflicts of interest.

## Data Availability

The data presented in this study is available on request from the corresponding author. The data are not publicly available due to legal issues.
